# Nerve identification during open inguinal hernia repair: a systematic review and meta-analyses

**DOI:** 10.1007/s00423-023-03154-2

**Published:** 2023-10-24

**Authors:** Viktor Bay Moseholm, Jason Joe Baker, Jacob Rosenberg

**Affiliations:** Center for Perioperative Optimization, Department of Surgery, Herlev Hospital, University of Copenhagen, Copenhagen, Denmark

**Keywords:** Nerves, Groin, Hernia, Pain, Surgery

## Abstract

**Purpose:**

Inguinal hernia repair is one of the most common operations worldwide and despite this, the incidence of chronic pain remains high after inguinal hernia repair. The optimal nerve handling strategy is controversial and the rate at which nerves are identified remains uncertain. This study aimed to determine the identification rates of the ilioinguinal, iliohypogastric, and genitofemoral nerves as well as nerve handling strategies.

**Methods:**

This review was registered on PROSPERO (CRD 42023416576). PubMed, Embase, and Cochrane Central were systematically searched. Studies with more than 10 patients were included if they reported an identification rate for at least one of the nerves during elective open inguinal hernia repair in adults. Studies requiring nerve identification in their study design were excluded. Bias was assessed with the JBI critical appraisal tool and Cochrane’s RoB-2 tool. The overall estimate of the prevalence was analysed with prevalence meta-analyses.

**Results:**

A total of 22 studies were included. The meta-analyses included 18 studies, which resulted in an identification rate of 82% (95% CI: 76–87%) for the ilioinguinal nerve, 62% (95% CI: 54–71%) for the iliohypogastric nerve, and 41% (95% CI: 27–55%) for the genitofemoral nerve. Nerves were spared in 82% of all repairs.

**Conclusion:**

The ilioinguinal, iliohypogastric, and genitofemoral nerves were identified in 82%, 62%, and 41% of surgeries, respectively. Most studies used a nerve-preserving strategy. The role of nerve identification in the development of chronic pain remains uncertain, as well as the optimal nerve handling strategy.

## Introduction

Inguinal hernia is a common surgical condition worldwide, with a lifetime incidence of 27 to 43% in men and 3 to 6% in women [1]. Although laparoscopic procedures have become more prevalent [2], open repairs are still commonly used, with the Lichtenstein technique being the most prevalent method [1]. Postoperative pain and sensory disturbances remain important complications of inguinal hernia repair, with an incidence of 10 to 63% of patients experiencing any pain and 1 to 18% experiencing moderate to severe pain that affects their daily lives [3]. However, these rates are highly debated and may be outdated [3]. It is assumed that the cause of pain is neurogenic in nature [4, 5]. During open inguinal hernia repair, the three nerves potentially encountered are the ilioinguinal, iliohypogastric, and genitofemoral nerves [6]. No consensus has been reached regarding whether to cut or preserve the encountered nerves, and the topic remains controversial. Studies have shown positive results for both strategies in terms of chronic pain and sensory disturbances [7, 8]. Other studies have concluded that nerve identification is important for positive patient outcomes, although this too remains uncertain [6, 8, 9]. However, data from a real-life setting where surgeons do not actively search for the nerves are sparse.

The aim of this study was to investigate the identification rates of the ilioinguinal, iliohypogastric, and genitofemoral nerves during elective open inguinal hernia repair, among studies that did not actively search for them. Secondly, we will present preservation and neurectomy rates, and the incidence of chronic pain.

## Methods

This systematic review was reported according to the Preferred Reporting Items for Systematic Reviews and Meta-Analyses (PRISMA) guidelines [10]. A protocol was preregistered on PROSPERO (CRD 42023416576) before the study inclusion process began. Studies were included from the following inclusion criteria: elective surgery for open inguinal hernia repair that reported an identification rate of any of the three nerves in adult patients (≥18 years old), which had a sample size of more than 10 patients. Studies were excluded if more than 10% of procedures were acute repairs or if they were cadaver studies. Randomised controlled trials (RCTs) and cohort studies with nerve identification or management as the primary outcome were also excluded as they would not represent common practice in regard to nerve identification. Studies stating that nerve identification during surgery was required per protocol were likewise excluded. Generally, studies had to be representative of common practice in the institution in which the study was performed.

### Search strategy and selection process

PubMed (from 1966 to present), Embase (through Ovid, from 1974 to present), and Cochrane Central were systematically searched. The search string was developed in cooperation with an information specialist. The search string for PubMed was: ((peripheral nerves [Mesh] OR ilioinguinal* OR genitofemoral* OR iliohypogastric* OR nerve*) AND (hernia, inguinal [mesh] OR (hernia AND (inguinal OR groin))). The search string was adapted to Embase and Cochrane Central. Moreover, forward and backward citation searches were also conducted for the included studies to further strengthen our search. No language restrictions or restrictions on publication date were used. Titles and abstracts were compiled into the Covidence screening software [11], which conducted automatic removal of duplicates. Article titles and abstracts were independently screened by two authors and any disagreements were resolved through discussion. This was followed by full-text screening, forward and backward citation search, and data extraction.

### Data items and data extraction

Data items included general study characteristics as well as number of operations, nerve identification and handling, surgical technique, and incidence of chronic pain or sensory disturbances ≥3 months after surgery. Articles not in English or Danish were translated using ChatGPT [12].

### Bias assessment

The Joanna-Briggs Institute (JBI) critical appraisal tool for observational studies [13] was used for bias assessment of observational studies. We removed question 8 (*“Was there appropriate statistical analysis?”*) in our bias assessment since it was not applicable to our main outcome. For randomised controlled trials, we used the Cochrane Risk of Bias-2 (RoB-2) tool [14].

### Statistical analysis

Meta-analysis was conducted to analyse the prevalence of identification for each nerve separately. Subgroup analyses were conducted excluding studies with a high risk of bias or with clinical heterogeneity. This study investigated the frequency of nerve identification, and its ability to link it directly to chronic pain is therefore reduced. Given the variability in study conditions and other factors, making definitive associations is challenging, and, therefore, we will not conduct any meta-analyses or infer causal relationships. We used the OpenMeta[Analyst] software for the synthesis of the meta-analysis of an untransformed proportion, using the “meta-analysis” function [15], and forest plots for visualisation of results. Results were graded using the GRADE tool [16].

## Results

**Fig. 1:**
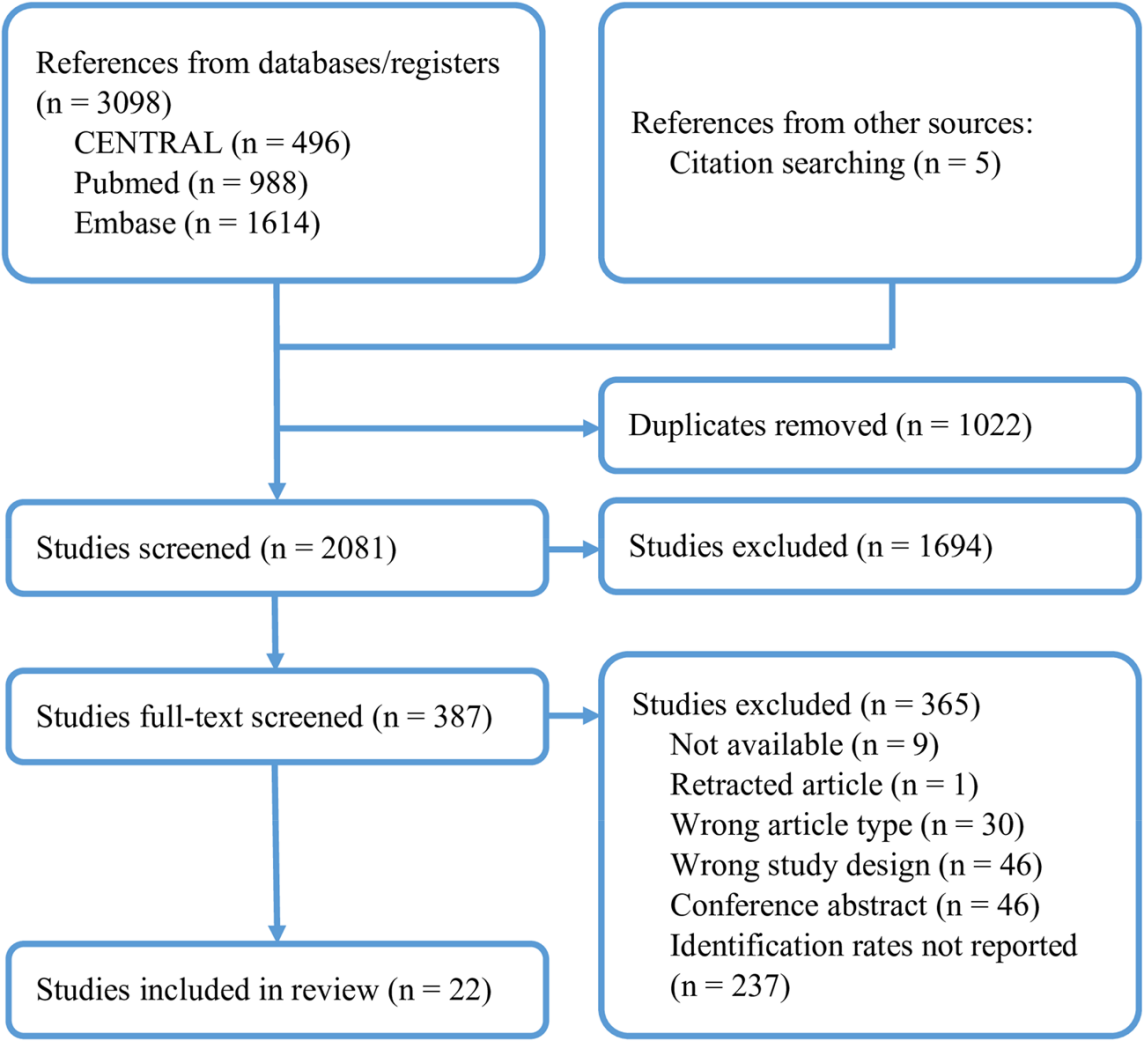
PRISMA flowchart of included studies. *n* = number of studies

The PRISMA flowchart of the study selection process is presented in Fig. 1. Initially, 3098 articles were identified and a total of 387 articles were eligible for full-text screening, from which 17 articles were included [6, 8, 17–31]. Furthermore, five additional articles were identified from forward and backward citation searches [32–36], resulting in a total of 22 studies included in our analyses. However, three studies reported on the same population [17, 23, 35], and only the study that provided the clearest information regarding nerve identification was analysed [17]. Similarly, two other studies had overlapping populations [24, 34], and only the original study was included in the analyses [34]. Thus, 18 studies [6, 8, 17–21, 25–34, 36] were included in the meta-analyses.

### Summary and characteristics of included studies

The included studies comprised a total of 28,481 groins (Table 1), with eight RCTs and 11 cohort studies. The median age of the participants across the studies was 58 years (range 52–69 years). The surgical technique was comparable between studies with the Lichtenstein technique used in 74% of the studies and all but one study [27] used mesh in all repairs. This study used the Lichtenstein technique in approximately 2/3 of operations and the Shouldice technique in 1/3 of operations [27].

**Table 1:** Study characteristics

			*Identification*			*Preservation*		
*Study*	Type	Groins	IIN %	IHN %	GFN %	IIN %	IHN %	GFN %
O’Dwyer 2005 [34]	RCT	316	92.1	43.0	47.8	78.0	84.6	80.8
Alfieri 2006 [8]	Cohort	973	70.8	59.0	55.6	84.5	89.5	88.7
Bartlett 2007 [24]	Cohort	172	97.7	85.5	86.1	80.4	90.5	91.9
Nienhuijs 2007 [25]	RCT	86	77.9	26.7	24.4	92.5	87.0	95.2
Magnusson 2010 [26]	Cohort	70	75.7	-	-	67.9	-	-
Smeds 2010 [6]	Cohort	525	73.3	64.8	13.5	75.8	52.1	81.7
Reinpold 2011 [27]	Cohort	781	88.0	45.3	38.2	85.5	88.0	93.5
Sadowski 2011 [28]	RCT	78	93.6	55.1	16.7	-	-	-
Bischoff 2012 [29]	Cohort	244	97.5	94.7	21.9	-	-	-
Campanelli 2012 [30]	RCT	316	90.2	84.8	71.8	86.7*	84.3*	92.1*
Kingsnorth 2012 [35]	RCT	302	91.7	65.9	53.0	-	-	-
Hirose 2013 [31]	RCT	182	93.4	89.6	95.6	98.2	96.9	98.9
Jorgensen 2013 [33]	RCT	334	92.2	71.9	21.9	-	-	-
Ruiz-Jasbon 2014 [36]	Cohort	39	66.7	15.4	20.5	92.3	83.3	100.0
Sanders 2014 [17]	RCT	557	88.3	66.3	47.4	71.1	66.4	90.5
Azeem 2015 [18]	RCT	42	35.7	73.8	23.8	-	-	-
Smeds 2016 [23]	Cohort	507	89.0	68.0	48.0	70.1	64.6	92.2
Wright 2019 [19]	Cohort	143	86.0	-	-	0.0	-	-
Cirocchi 2020 [20]	Cohort	115	82.6	72.2	48.7	0.0	-	-
Melkemichel 2020 [21]	Cohort	23,259	75.0	55.6	21.9	85.8	86.2	79.9
Farquharson 2021 [22]	Cohort	100	9.0	1.0	2.0	22.2	0.0	0.0
Hara 2021 [32]	Cohort	321	69.2	73.2	85.4	82.0	33.6	93.1

All preservation rates are calculated from the total number of nerve identifications. *n*, amount; *Res. Q.*, research question; *IIN*, ilioinguinal nerve; *IHN*, iliohypogastric nerve; *GFN*, genitofemoral nerve. *Calculated under the assumption that all nerves not divided have been preserved

Regarding the identification rates, 19 studies reported on the identification rates of the ilioinguinal nerve, and 17 studies reported on the iliohypogastric and genitofemoral nerves. The crude identification rates were as follows: the ilioinguinal nerve was identified in 76% of repairs (range 0–98%), the iliohypogastric nerve was identified in 57% of repairs (range 1–95%), and the genitofemoral nerve was identified in 25% of cases (range 2–96%).

Most studies used a nerve-sparing technique. Out of the identified nerves, the ilioinguinal nerve was preserved in 83% of the repairs (range 0–98%), the iliohypogastric nerve was preserved in 83% of the repairs (range 0–97%), and the genitofemoral nerve was preserved in 82% of the repairs (range 0–98%).

The crude rate of neurectomy was comparable between the nerves. The ilioinguinal nerve was resected in 15% of the repairs (range 7–100%), the iliohypogastric nerve was resected in 15% (range 1–66%), and the genitofemoral nerve was resected in 17% of the repairs (range 0–20%).

Chronic pain was reported with different follow-up periods, ranging from 3 to 41% at three months, and between 2 and 22% at 12 months. Only four studies reported on sensory disturbances, with incidences ranging from 33 to 36% at 3 months [18], 15 to 16% at 6 months [20, 27], and 22% at 12 months [26]. No studies reported on hyperesthesia.

### Bias assessment

**Fig. 2:**
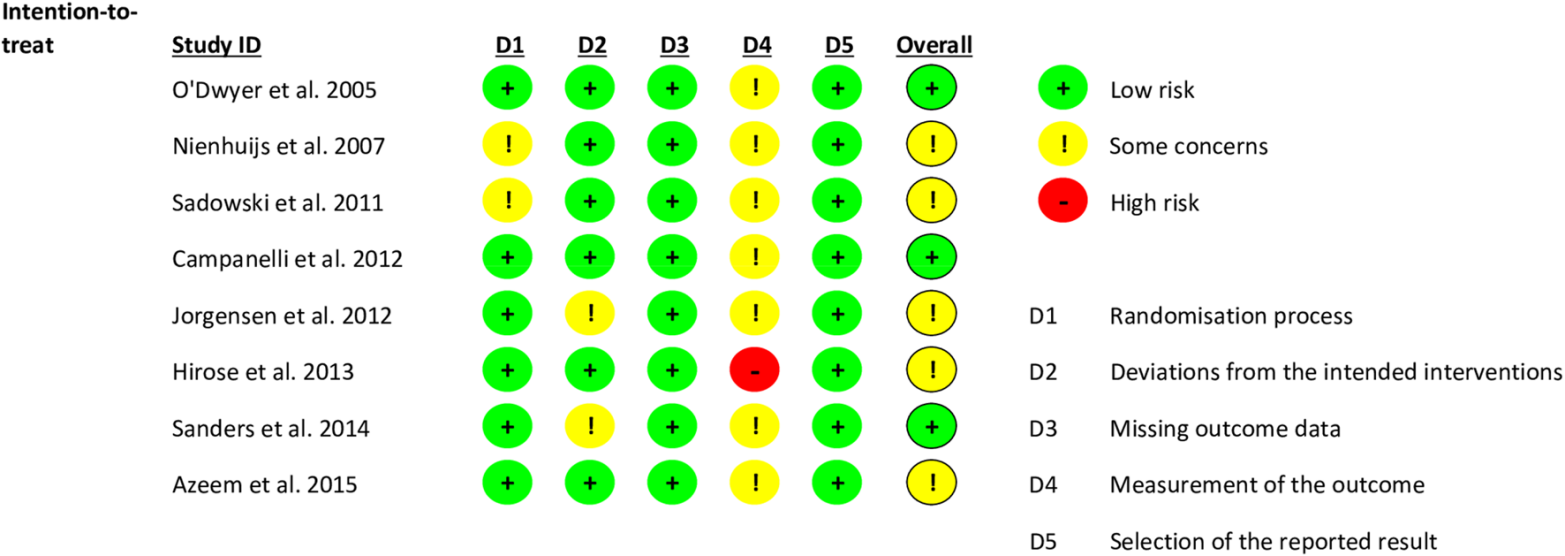
Risk of bias assessment for randomised controlled trials (RCTs) included in this study using the Cochrane’s risk of bias 2 assessment tool

Figure 2 presents the results of the bias assessment of included RCTs. In the second domain, all RCTs were rated as having “some concerns” overall. This was because no study clearly stated whether nerves were identified according to common practice at the given institution. Hirose et al. [31] were rated as “high risk” in the second domain because it was unclear whether nerve identification was part of their study protocol.

Table 2 displays the results of the bias assessment of the observational studies using the JBI critical appraisal tool. Overall, four studies [6, 22, 26, 27] had a moderate risk of bias in question 4, because it was unclear whether nerves were identified according to common practice. These studies were excluded from the sensitivity analyses, which excluded studies with a moderate to high risk of bias. Only two cohort studies reported that nerve identification was performed by an experienced surgeon [27, 29]. The rest of the observational studies did not adequately report the surgical experience of the operating surgeon, as reflected in question 7 of the JBI critical appraisal tool (defined as being mentioned as “experienced,” “trained,” “expert,” or “single surgeon”). Regarding the response rate, Smeds et al. [6] had a response rate of 55% and did not account for non-responders at follow-up, but we assessed that this would not skew identification rates. Similarly, Farquharson et al. [22] noted that the majority of medical journal notes from which data were sampled did not mention any nerves, and was therefore excluded from the meta-analyses.

**Table 2:** JBI critical appraisal tool assessment

*Study*	Q1	Q2	*Q3*	*Q4*	*Q5*	*Q6*	*Q7*	*Q9*
Alfieri 2006 [8]	Yes	Yes	Yes	Yes	Yes	Yes	Unclear	Yes
Magnusson 2010 [26]	Yes	Yes	Yes	Yes	Yes	Unclear	Unclear	Yes
Smeds 2010 [6]	Yes	Yes	Yes	Yes	Yes	Yes	Unclear	No
Reinpold 2011 [27]	Yes	Yes	Yes	Yes	No	Yes	Yes	Unclear
Bischoff 2012 [29]	Yes	Unclear	Yes	No	Yes	Unclear	Yes	Yes
Ruiz-Jasbon 2014 [36]	Yes	Unclear	Yes	Yes	Yes	Yes	Unclear	Yes
Wright 2019 [19]	Yes	Yes	Yes	No	Yes	Unclear	Unclear	Yes
Cirocchi 2020 [20]	Yes	Unclear	Yes	Yes	Yes	Unclear	Unclear	Yes
Melkemichel 2020 [21]	Yes	Yes	Yes	Yes	Yes	Yes	Unclear	Yes
Farquharson 2021 [22]	Yes	Yes	Yes	Yes	Yes	Yes	No	No
Hara 2021 [32]	Yes	Yes	Yes	Yes	Yes	Yes	Unclear	Yes

The Joanna-Briggs Institute (JBI) critical appraisal tool used on identified cohort studies. Q1: Was the sample frame appropriate to address the target population? Q2: Were study participants sampled in an appropriate way (It was considered appropriate if the study reported to have included all patients or patients at random)? Q3: Was the sample size adequate? Q4: Were the study subjects and the setting described in detail? Q5: Was the data analysis conducted with sufficient coverage of the identified samples? Q6: Were valid methods used for the identification of the condition (“The condition” was defined as “the identification of nerves” as that was the subject of interest in this study. To what lengths did the researchers go to identify the nerves? Yes = representative of common practice. Unclear = it is unclear or difficult to determine how thoroughly nerves were searched for)? Q7: Was the condition measured in a standard, reliable way for all participants (nerve identification was valid if the study mentioned how the data were obtained and who performed the identification (junior/senior/trained surgeon))? Q9: Was the response rate adequate, and if not, was the low response rate managed appropriately?

Because the data points included in our study were typically presented in a study or population characteristics section, they have likely not influenced the publication bias. Therefore, we assessed that the reporting biases in this study were low.

### Results from meta-analyses

**Fig. 3:**
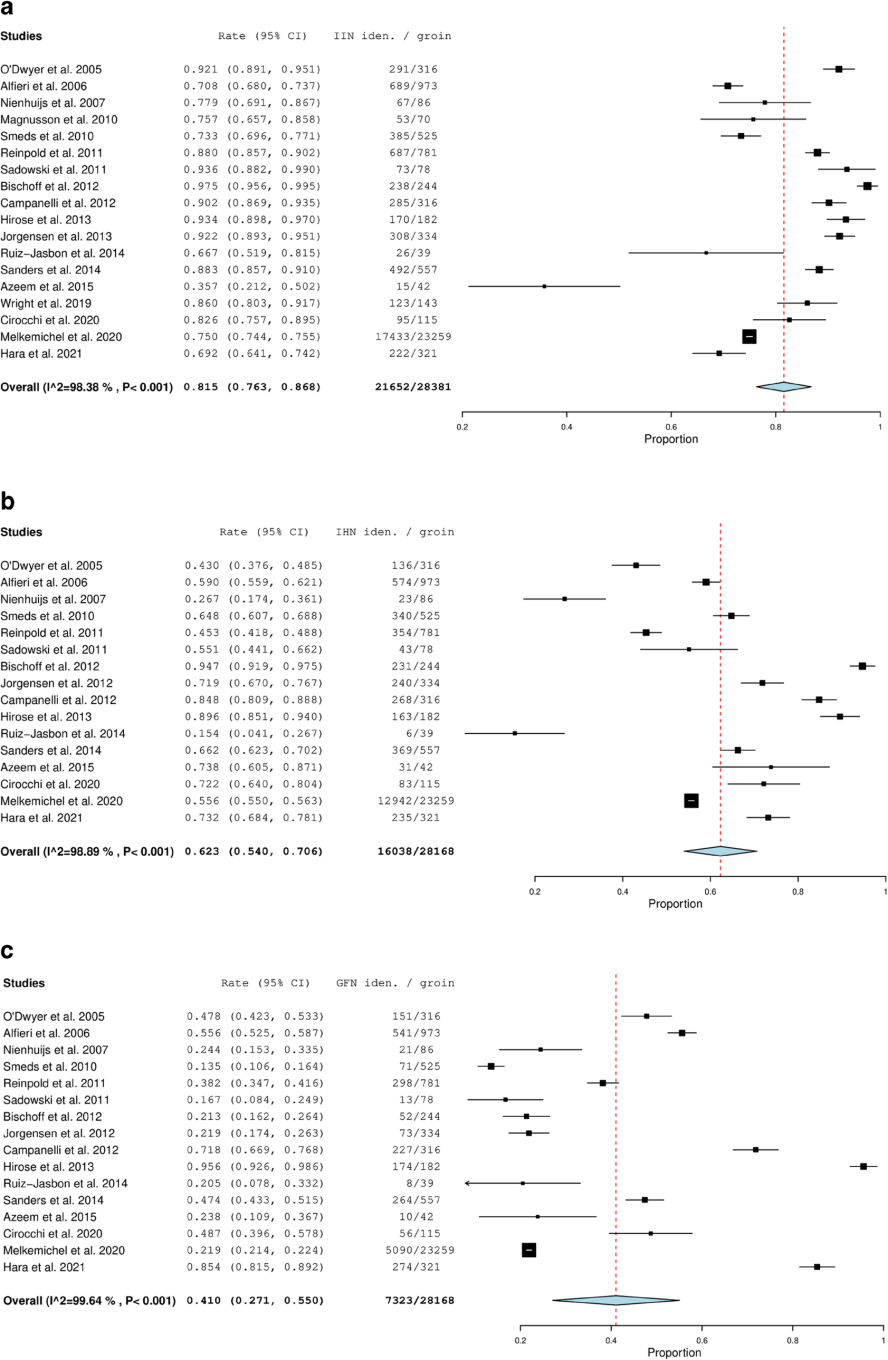
**a** Prevalence meta-analysis of the identification rates for the ilioinguinal nerve. **b** Prevalence meta-analysis of the identification rates for the iliohypogastric nerve. **c** Prevalence meta-analysis of the identification rates for the genitofemoral nerve. CI, confidence interval

The results from the meta-analyses of the pooled prevalences of nerve identification are presented in Fig. 3. Overall, the ilioinguinal nerve was the most frequently identified nerve, with an identification rate of 82% (Fig. 3a), which was significantly more than the two other nerves. The iliohypogastric nerve had an identification rate of 62% (Fig. 3b), while the genitofemoral nerve had an identification rate of 41% (Fig. 3c).

After removing studies with moderate to high risk of bias related to reporting of identification rates [19, 20, 26, 29, 31], the pooled prevalence of nerve identification declined nominally across all three nerves. However, the confidence intervals between the sensitivity analyses and main analyses overlapped, thus making these findings insignificant. The difference between nerves remained significant, with the ilioinguinal nerve being the most consistently identified nerve. The reductions were approximately 9% for the ilioinguinal nerve, 5% for the iliohypogastric nerve and 6% for the genitofemoral nerve. In a sensitivity analysis that excluded studies with expert surgeons [17, 19, 27, 29–31, 33, 36], the identification rate of the ilioinguinal nerve was nominally 6% lower than our main pooled estimate, with a prevalence of 76% (95% CI: 71–82%). Again, this difference was statistically insignificant, due to overlapping confidence intervals. The difference between nerves remained significant, with the ilioinguinal nerve being the most consistently identified nerve.

We used the GRADE tool to evaluate the quality of the evidence presented in this study, and we judged it to be of moderate quality. This means that we are moderately confident that the true identification rates are likely to be close to the estimates presented in our meta-analysis. However, there is a possibility that they may be substantially different from ours, due to the formerly discussed bias of the included studies. Our evidence quality started as high but was downgraded due to inconsistencies in reporting common nerve handling practices between studies.

## Discussion

In this study, we conducted a systematic review of the identification rates of three nerves present in the surgical field during open inguinal hernia repair. The ilioinguinal nerve was identified most frequently, and the prevalence meta-analysis showed an identification rate of 82%. In comparison, the identification rates of the iliohypogastric and genitofemoral nerves were 62% and 41%, respectively. The difference can be attributed to the anatomical location of the ilioinguinal nerve in the operative field, where it is usually observed running parallel to the spermatic cord after the fascia is opened, thus making it visible in the centre of the operative field. In contrast, the other two nerves typically require further dissection to be identified. The low identification rate of the genitofemoral nerve can be attributed to its usual dorsolateral course behind the spermatic cord. As one study has pointed out, dissection in this direction is not recommended, due to the risk of iatrogenic injury to the external spermatic vein [29]. We also observed significant variation in the way studies reported nerve identification rates. Nerves were more frequently identified in RCTs, likely due to their more standardised approach. Additionally, experienced surgeons were more likely to identify the ilioinguinal nerve, although by a small margin. The nerve-sparing approach remains the recommended approach [1, 37], which our analysis reflects, with the majority of identified nerves being spared. However, pragmatic nerve resection is still recommended [1, 37], and has been shown to decrease postoperative pain [6]. Pragmatic nerve resection is defined as the resection of nerves at risk of interfering with implanted mesh, risk of being damaged during surgery, or if nerves are already damaged during the dissection.

To our knowledge, this is the largest systematic review and meta-analysis on the prevalence of identification of the ilioinguinal nerve, the iliohypogastric nerve, and the genitofemoral nerve, in patients undergoing open inguinal hernia repair. Previous systematic reviews have investigated the identification rates of these three nerves [38, 39], reporting higher overall identification rates: 94 and 84% for the ilioinguinal nerve, 87 and 71% for the iliohypogastric nerve, and 69 and 53% for the genitofemoral nerve, respectively. However, these studies have methodological issues and may not accurately represent identification rates. Notably, these studies included RCTs that required nerve identification per protocol [38, 39], included cadaveric studies in their analysis [38], and included two studies reporting on the same population [38]. Our study is strengthened by not having any language or year restrictions, which yielded studies that may not have been otherwise identified. Another strength of our study is the rigorous systematic review methodology used, including forward and backward citation searches [40]. However, this study also has some limitations. It was difficult to discern the extent to which surgeons were actively looking for nerves in the included studies but we extensively investigated protocols where available and performed sensitivity analyses to adjust for this. There was also significant clinical heterogeneity between study designs. Additionally, determining the incidence of chronic pain after open inguinal hernia repair was challenging, as studies had different follow-up periods. Lastly, we cannot rule out some confounding by indication in our study. In institutions where reporting identified nerves during surgery is mandatory or routine, surgeons may automatically be more attentive to nerve identification.

This study provides valuable insights into the prevalence of identification of the ilioinguinal, iliohypogastric, and genitofemoral nerves during open inguinal hernia repair. The findings suggest that reporting of nerve identification rates varies greatly among studies. Hopefully, these results can shape future practice in the identification, and documentation of identification, of nerves. While several studies recommend that all nerves encountered during surgery should be confidently identified, it is unclear whether this has a significant impact on chronic postoperative pain [6, 8, 9, 37]. Some studies have shown a significant difference [6, 8], while others have not [23, 29]. In this study, we did not find an association between nerve handling and the incidence of chronic pain. The data on chronic pain or sensory disturbances did not permit meta-analysis due to heterogeneity. Nerve identification is feasible, as one study has shown that proper identification of all three nerves does not impede the surgical procedure significantly [41]. Overall, larger observational studies with standardised documentation and low risk of bias regarding nerve identification are needed to determine the potential impact on chronic pain and sensory disturbances. Institutions can use the identification rates presented in this study as a benchmark for the quality assessment of their own practices. Moving forward, future research on chronic pain and sensory disturbances will hopefully benefit from more uniform and precise reporting of nerve identification and handling during open inguinal hernia repair.

In conclusion, the identification rates for the three different nerves during open repair for inguinal hernias as reported in the literature were 82% for the ilioinguinal nerve, 62% for the iliohypogastric nerve, and 41% for the genitofemoral nerve. Most studies adopted a nerve-preserving strategy, with more than 82% of nerves being spared during surgery. Chronic pain rates varied between 4 and 41%, depending on follow-up. Further research is needed to ascertain the role of nerve identification on patient outcomes and whether nerves should be preserved or resected.
