# Correction to: Nerve identifcation during open inguinal hernia repair: a systematic review and meta-analyses

**DOI:** 10.1007/s00423-023-03180-0

**Published:** 2023-11-18

**Authors:** Viktor Bay Moseholm, Jason Joe Baker, Jacob Rosenberg

**Affiliations:** Center for Perioperative Optimization, Department of Surgery, Herlev Hospital, University of Copenhagen, Copenhagen, Denmark

**Correction to: Langenbeck's Archives of Surgery**

10.1007/s00423-023-03154-2

In the results section of the abstract the ‘23’ in the sentence "A total of 23 studies were included." should be changed to 22, so that it reads: "A total of 22 studies were included."

The first line of the result sections has been corrected.

The original article has been corrected.

